# Mining microbial and metabolic dark matter in extreme environments: a roadmap for harnessing the power of multi-omics data

**DOI:** 10.1007/s44307-024-00034-8

**Published:** 2024-08-05

**Authors:** Jia-Rui Han, Shuai Li, Wen-Jun Li, Lei Dong

**Affiliations:** 1 State Key Laboratory of Biocontrol, Guangdong Provincial Key Laboratory of Plant Stress Biology and Southern Marine Science and Engineering Guangdong Laboratory (Zhuhai), School of Life Sciences, Sun Yat-Sen University, Guangzhou, 510275 PR China; 2 State Key Laboratory of Desert and Oasis Ecology, Xinjiang Key Laboratory of Biodiversity Conservation and Application in Arid Lands, Xinjiang Institute of Ecology and Geography, Chinese Academy of Sciences, Urumqi, 830011 PR China

**Keywords:** Extrem environments, Multi-omics, Microbial dark matter, Metabolic dark matter, Biosynthesis

## Abstract

Extreme environments such as hyperarid, hypersaline, hyperthermal environments, and the deep sea harbor diverse microbial communities, which are specially adapted to extreme conditions and are known as extremophiles. These extremophilic organisms have developed unique survival strategies, making them ideal models for studying microbial diversity, evolution, and adaptation to adversity. They also play critical roles in biogeochemical cycles. Additionally, extremophiles often produce novel bioactive compounds in response to corresponding challenging environments. Recent advances in technologies, including genomic sequencing and untargeted metabolomic analysis, have significantly enhanced our understanding of microbial diversity, ecology, evolution, and the genetic and physiological characteristics in extremophiles. The integration of advanced multi-omics technologies into culture-dependent research has notably improved the efficiency, providing valuable insights into the physiological functions and biosynthetic capacities of extremophiles. The vast untapped microbial resources in extreme environments present substantial opportunities for discovering novel natural products and advancing our knowledge of microbial ecology and evolution. This review highlights the current research status on extremophilic microbiomes, focusing on microbial diversity, ecological roles, isolation and cultivation strategies, and the exploration of their biosynthetic potential. Moreover, we emphasize the importance and potential of discovering more strain resources and metabolites, which would be boosted greatly by harnessing the power of multi-omics data.

## Introduction

**Fig. 1:**
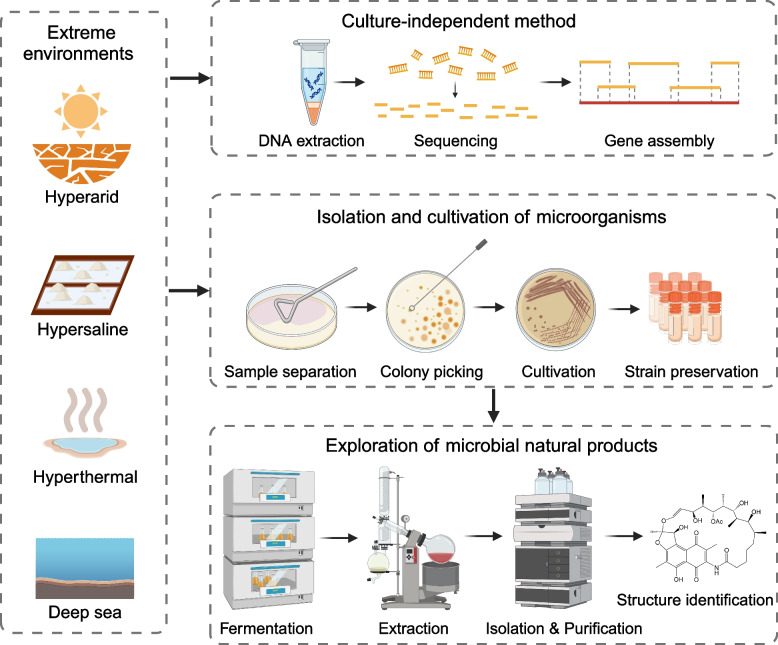
Primary approach to understanding and exploring microbial resources form extreme environments

Extreme environments are prevalent globally, including hyperarid, hypersaline, hyperthermal environments, and the deep sea (Rothschild and Mancinelli 2001; Schmid et al. 2020). Despite the harsh conditions in these habitats, they provide diverse ecological niches for various microorganisms (Shu and Huang 2022). These organisms have evolved various strategies such as altering lipid compositions, producing specific proteins, and adjusting ion concentrations to thrive in extreme environments (Gunde-Cimerman et al. 2018; Lewin et al. 2013). This presents an ideal research model for investigating the physiological limits of microbial survival, evolution, environmental adaptation, and their roles in biogeochemical cycling. Moreover, in adapting to the extreme conditions of their environments and competing for limited resources in nutrient-poor habitats, microorganisms often produce novel and bioactive natural products (van Bergeijk et al. 2020). Since these metabolites are also largely unknown, we refer to them as “metabolic dark matter”. Therefore, in this review, we provide a comprehensive summary of the current research on extremophilic microbiomes, detailing empirical approaches for isolation and cultivation methods, and propose the rational pathway for exploring biosynthetic potential by integrative multi-omics study. We outline a detailed roadmap for deep mining of extremophiles to better excavate the genetic resources in uncultured microbial dark matter, enhance the possibility from concept to the cultivation of targeted taxa, and effectively access their metabolic potential (Fig. 1).

## Microbiome of extreme environments

The characteristics of extreme environments, include extreme temperature or pH values, high salinity stress, low levels of nutrients, and specific combinations, posing significant challenges to life survival (Rothschild and Mancinelli 2001). With the advancement and widespread adoption of DNA sequencing technologies, microbiologists have discovered abundant microbial resources in extreme environments. Many previously unknown microbial lineages, so called the microbial dark matter, have been revealed by diversity surveys in extreme environments. However, obtaining pure cultures for most of these microbes remains a challenge, which impedes further exploration of their physiological and ecological functions (Hedlund et al. 2015). Nevertheless, metagenomics and single-cell genomics technologies enable the reconstruction of near-complete microbial genomes directly from the environment, which could provide deeper insights into the metabolic potentials and evolutionary history of these uncultivated extremophilic microorganisms (Tyson et al. 2004). Furthermore, the relatively lower biological complexity, and tight coupling between geochemical and biological processes, renders some extreme environments ideal research models for studying microbial ecology, evolution, overall tractability for cultivation-independent molecular analyses and environmental adaptation (Shu and Huang 2022). In this section, we reviewed the extremophilic microbiomes of four distinct extreme environments : hyperarid, hypersaline, hyperthermal environments, and the deep sea, which are the hotspots in recent decades.

### Hyperarid environment

The desert ecosystem represents hyperarid environments and constitute the largest terrestrial ecosystem on Earth, covering approximately one-third of the global land surface (Neilson et al. 2017). Desert microbes play a crucial role in maintaining ecological stability and biogeochemical cycles. Analyzing the microbial biodiversity, composition, and functions within desert ecosystems can help understand the threats and opportunities brought by global change and arid land life zones (Arora and Panosyan 2019).

Aridity, defined as the ratio of precipitation to potential evapotranspiration, plays a predominant role in shaping the diversity and composition of microbial communities in global arid land soils (Maestre et al. 2015). Specifically, increased aridity reduces plant cover and soil organic carbon content, consequently significantly decreasing microbial diversity and abundance (Maestre et al. 2015). Nonetheless, desert ecosystems still harbor diverse bacterial lineages such as the phyla *Actinomycetota*, *Chloroflexota*, *Bacillota*, *Gemmatimonadota*, and *Pseudomonadota*. A recent global survey indicated that the phylum *Actinomycetota* was the most abundant group detected in desert ecosystems at 25.5%, followed by the phylum *Pseudomonadota* (21%), *Acidobacteriota* (6.5%), *Verrucomicrobiota* (6%), *Chloroflexota* (2.5%), and *Bacillota* (2%) (Leung et al. 2020). Research in the Atacama Desert, Chile, revealed a significant positive correlation between community richness and diversity with relative soil moisture (Neilson et al. 2017). The strongest and most significant correlation between relative soil moisture and microbial abundance was observed in the phyla *Acidobacteriota*, *Pseudomonadota*, *Planctomycetota*, *Verrucomicrobiota*, and *Nitrosophaerota* (Neilson et al. 2017). The reduction in average relative soil moisture and increase in temperature explained the significant decrease in diversity and connectivity of desert soil microbial communities, leading to a notable decrease in the abundance of key taxa typically associated with fertile soils (Neilson et al. 2017). Studying the diversity and ecological functions of desert crop rhizosphere microbes in several desert farms in the Sinai Desert of Egypt reveals a high diversity of microbial groups (Lian et al. 2023). This demonstrated that environmental differences arising from geographical locations have a greater impact on rhizosphere microbial communities than the host plants themselves. Recent study on the microbiome of northwest Chinese desert soils categorized them into semi-arid (0.63 < aridity ≤0.8), arid (0.8 < aridity ≤0.97), and hyper-arid (aridity >0.97), finding that microbial diversity decreases with increasing aridity (Dong et al. 2024). However, the abundance of phylum *Bacillota* increases with higher aridity levels, suggesting this group may possess capabilities to withstand environmental stress conditions (Dong et al. 2024). Li et al. conducted a survey of culture-independent and culture-dependent diversity in samples from the Gurbantunggut Desert. It not only elucidated the species novelty of microbial communities but also found that culture-dependent methods serve as a valuable complement to culture-independent techniques. Additionally, they successfully isolated 1589 bacterial strains and 469 actinomycete strains, including numerous potential novel taxonomic units. These new isolated resources will be an essential basis for exploring and verifying the physiological and ecological functions of desert microbes, as well as for the developing and applying their cryptic biosynthetic potential, what we call metabolic dark matter.

### Hypersaline environment

In hypersaline environments such as salt lakes and solar salterns, microbial community structures undergo significant shifts along salt concentration gradients, with archaea dominating at the highest salt concentrations. The Santa Pola multipond solar saltern in Alicante, Spain, exhibited a wide range of salt concentrations. Studies on the diversity reveal that in most saturated brines, the square archaeon *Haloquadratum walsbyi* and the bacteroidete *Salinibacter ruber* are predominant in prokaryotic microbial diversity, whereas greater bacterial and archaeal diversity is observed under moderate salinity conditions (Ventosa et al. 2015). Lake Tyrrell in Victoria, Australia, is a hypersaline lake with significant seasonal variations in environmental conditions, particularly salt concentrations. The microbial community composition in Lake Tyrrell typically varies with time and space, with seasonal fluctuations in ion concentration being a major driver of microbial succession (Emerson et al. 2013; Podell et al. 2013).

Moreover, extensive benthic microbial mats thrive in salt field evaporation ponds have been observed highly stratified due to physiochemical gradients, especially light, oxygen, and sulfide gradients (Oren 2015). Extensive 16S rRNA gene sequencing of deep profiles of high-salt mats in the Guerrero Negro, Mexico, revealed it to be one of the most diverse and complex known environments phylogenetically, uncovering several new phylum-level bacterial groups and numerous previously undetected lower-level taxonomic groups (Kirk Harris et al. 2012). *De novo* metagenomic assembly of multiple libraries from surface water in Lake Tyrrell revealed *Ca.* Nanohaloarchaeota within the superphylum DPANN (Narasingarao et al. 2011). These ultra-small but metabolically diverse halophilic microbes are widely distributed in high-salt environments worldwide (Narasingarao et al. 2011). Co-cultivation of Antarctic *Ca.* Nanohaloarchaeota strains has suggested host dependency in these halophilic archaea (Hamm et al. 2019). In Qi Jiao Jing (QJJ) Lake in Xinjiang Uyghur Autonomous Region, China, a novel taxon of *Ca.* Nanohaloarchaeota, *Nucleotidisoterales*, has been discovered (Xie et al. 2022). Unlike other *Ca.* Nanohaloarchaeota, *Ca.* Nucleotidisoterales is incapable of degrading polysaccharides. Instead, genomic analyses indicated their ability to salvage and degrade nucleotides and proteins for synthetic metabolism and energy conservation, suggesting their occupation of a distinct ecological niche. Phylogenetic investigations suggested that *Nucleotidisoterales* may serve as a donor of *rbcL* genes for *Halobacteria*.

### Hyperthermal environment

In the global studies, phyla *Aquificota*, *Pseudomonadota*, and *Crenarchaeota* typically dominate in terrestrial geothermal hot springs (Cole et al. 2012; Power et al. 2018). Significant taxa include carbon-assimilating *Hydrogenobaculum* spp., sulfur-oxidizing *Thermoproteus* spp. and *Sulfolobus* spp., and nitrogen-fixing *Acidithiobacillus* spp., with marked differences existing between microbial communities in the same hot spring water and sediment (Cole et al. 2012; Colman et al. 2016a). Sediment communities exhibited relatively higher species evenness and are predominantly composed of taxa capable of mineral metabolism such as sulfur oxidation, reduction, or iron oxidation-reduction (Cole et al. 2012; Colman et al. 2016a). Microbial mats thriving in hot springs generally show low microbial diversity, with dominant taxa being phyla *Cyanobacteriota* (mostly *Synechococcus* spp.) and *Chloroflexota* (*Roseiflexus* and *Chloroflexus* spp.) (Miller et al. 2009). Multiple studies indicated that temperature is a predominant factor influencing community composition in hot spring ecosystems at different temporal and spatial scales (Cole et al. 2012; Miller et al. 2009; Sharp et al. 2014). Metagenomic sequencing and genome assembly have also revealed a wide array of uncultivated bacterial lineages present in hot spring ecosystems, including phyla *Ca.* Acetothermota, *Ca.* Fervidibacterota, *Ca.* Calescibacteriota, *Ca.* Atribacterota, and *Ca.* Caldipriscota (Colman et al. 2016b; Rinke et al. 2013; Takami et al. 2012). Surveying a massive global set of metagenomic data for novel microbial lineages has led to the discovery of phylum *Ca.* Kryptoniota in high-temperature circumneutral pH geothermal springs (Eloe-Fadrosh et al. 2016). The phylum *Ca.* Atribacterota is widely distributed across various anaerobic environments. Through metagenomic assembly and single-cell genome analysis, this group has been identified as heterotrophic anaerobic microorganisms lacking respiratory capability, with fermentation and syntrophy as common physiological traits, indicating their significant role in the carbon cycle in anoxic environments. Genomic analysis of the phylum *Ca.* Kryptoniota has also identified its unique metabolic pathways and distinct heterotrophic lifestyle with nutritional deficiencies, hinting at metabolic collaborations with other groups such as the *Armatimonadetes* lineage to complement missing metabolic features.

Hot spring ecosystems also harbor diverse archaeal taxa. Research by Hua et al. indicated that the Xizang and Yunnan hot spring contains rich microbial resources (Hua et al. 2019). Using metagenomic sequencing techniques, they reconstructed 14 microbial genomes with methane/alkane metabolism capabilities, showcasing exceptionally high and unique phylogenetic diversity. These genomes are widely distributed within the *Verstraetearchaeota*, *Nezharchaeota*, and other TACK superphylum. Additionally, methane-producing functions were discovered within the phylum *Thaumarchaeota*. In sediment from the Tengchong hot springs in China, complete genomes of six uncultivated microbial groups belonging to the phylum *Aigarchaeota* were reconstructed (Hua et al. 2018). Analysis revealed that they possess strict or facultative anaerobic lifestyles and the ability to oxidize sulfides to conserve energy.

### Deep sea

In the deep sea, energy limitations are typically extreme. Without the introduction of new energy sources from sunlit oceans, microbes may remain in an energy-limited state for extended periods, sometimes up to millions of years (Hoehler and Jørgensen 2013). Under such resource-restricted conditions, interspecies interactions such as metabolic cross-feeding and complementation in biosynthesis play a crucial role in enabling microbial communities to fully utilize energy and growth substrates (Nawaz et al. 2022). Microbes in the deep sea transduce energy through biochemical catalysis of redox reactions, utilizing electron donors including methane, hydrogen, reduced iron, reduced manganese, reduced sulfur, ammonia, and ammonium (Bach and Edwards 2003). Electron acceptors in the deep sea include oxygen, oxidized nitrogen compounds like nitrate and nitrite, oxides of manganese and iron, oxidized sulfur compounds like sulfate and sulfite, and oxidized carbon compounds like carbon dioxide (Bradley et al. 2020). The availability of various electron donors and acceptors in various deep biosphere habitats is a key driver shaping microbial community diversity, ecological functions, and biogeography (Graw et al. 2018).

Deep-sea sediments are primarily classified as oxic and anoxic, exhibiting distinct microbial diversities (Orsi 2018). Anoxic sediment cells outnumber oxic sediment cells by several orders of magnitude and are rich in strict anaerobic bacterial groups such as sulfate-reducing bacteria, anaerobic members of the phylum *Chloroflexota*, methanogens and methane-consuming archaea, members of the phylum *Ca.* Atribacterota, and fermentative archaea (Orsi 2018). In contrast, oxic sediments harbor different communities composed of obligate aerobic and facultative anaerobic heterotrophs belonging to the phyla *Pseudomonadota* and *Chloroflexota*, Marine Group II (MG-II) archaea, and chemolithoautotrophic members of the phylum *Thaumarchaeota* (Danovaro et al. 2016; Durbin and Teske 2011; Walsh et al. 2015). Although the absolute abundance of cells in anoxic sediments surpasses that of oxic sediments by orders of magnitude, the ratio of archaea to bacteria is similar in both oxic and anoxic sediments (Lloyd et al. 2013).

Deep-sea bacteria are categorized based on their optimal growth pressures into piezotolerant bacteria (growing at pressures of 0.1-10 MPa), piezophiles (growing at pressures of 10-70 MPa), and super-piezophiles (not growing at pressures below 50 MPa) (Fang and Bazylinski 2008). Piezotolerant bacteria exhibit a relatively high diversity and less stringent pressure requirements (Zhang et al. 2018). Piezophiles can be cultured but display lower diversity, primarily represented by five bacterial genera within the class *Gammaproteobacteria*, including *Colwellia*, *Photobacterium*, *Psychromonas*, *Shewanella* and *Moritella* (Oger and Cario 2014). Most piezophilic archaea are (hyper-) thermophiles from the order *Thermococcales* (Oger and Cario 2014). Obligate piezophiles are constrained by unique temperature and pressure requirements and may be indigenous inhabitants of the deep sea, such as *Psychromonas hadalis*/*kaikoae*, *Colwellia hadaliensis*/*piezophila*, *Shewanella benthica*, and *Moritella yayanosii*. In contrast, some piezotolerant bacteria may originate from other environments, such as spore-forming bacteria like *Clostridium* spp. and *Bacillus stearothermophilus* (Bartholomew and Rittenberg 1949; Lauro et al. 2004; Zhang et al. 2018).

## Isolation and cultivation strategies for extremophilic prokaryotic microorganisms

Although genomic data can provide valuable insights into the importance and novelty of microorganisms, uncultivated lineages require the isolation and cultivation of representative strains to validate their cellular and physiological functions, thereby enabling a correct understanding of their ecological roles (Lewis et al. 2021). Li et al. found in a microbial diversity survey of desert ecosystems that amplicon analysis of the 16S rRNA gene revealed an extremely low abundance of Actinobacteria in the samples (0.04-0.37%, average 0.22%) (Li et al. 2021). In contrast, cultivation-dependent methods yielded a significantly higher abundance of Actinobacteria (5.2-39.6%, average 27.8%). The diversity of Actinobacteria obtained through cultivation-dependent methods far exceeded that from non-cultivation methods, highlighting the importance and indispensability of cultivation-dependent approaches in exploring microbial diversity. Additionally, natural products derived from microorganisms are also obtained through cultured isolates (Atanasov et al. 2021). Access to cultivable microbial strain resources is crucial for the in-depth exploration and application of their biosynthetic potential.

### Generate isolation and cultivation strategy

In cultivation-based studies, the effectiveness of isolation media is the key point. Mature isolation strategies often combine multiple selective culture media to obtain a richer diversity of isolated strains. The effectiveness of isolation media is closely linked to the ecological characteristics of target microbial groups. Successful cultivation of strains is intricately related to factors such as carbon and nitrogen sources, minerals, vitamins, moisture, and essential growth factors (Bonnet et al. 2020). Cultivation media are generally categorized as rich or minimal based on nutrient content. Research indicates that minimal media are more efficient in isolating Actinobacteria, creating a low-organic environment that is particularly selective for extremophiles (Bérdy, 2005; Bredholdt et al. 2007; Hozzein et al. 2008).

To enhance the isolation efficacy, researchers often introduce inhibitors into the isolation media. For instance, in studies focusing on Actinobacteria, particularly Gram-positive and Gram-negative bacteria, as well as fungi that compete with the target groups on isolation plates, are considered contaminants (Bonnet et al. 2020). Chemicals, such as antibiotics, are widely used to inhibit or eliminate these contaminants, ensuring the isolation ratio and efficiency of the targeted groups (Yousef and Carlstrom 2003). Commonly used inhibitors in *Streptomyces* isolation media include cycloheximide, nystatin, and nalidixic acid (Abdel-Aziz et al. 2021; Cong et al. 2019). Setting appropriate culture conditions is also crucial, with key factors including temperature and duration (Epstein 2013; Vieira and Nahas 2005). Given the diverse temperature preferences of microorganisms in extreme environments, selecting unconventional culture temperatures based on the research target can lead to increased isolation of rare microbial groups (Rego et al. 2019). Another factor is the culture duration, as setting specific durations can isolate groups with different growth rates, but this decision must consider various factors such as the type of isolation media, culture temperature, and physiological characteristics of target microbial groups.

The points discussed above are important factors influencing isolation cultivation studies. Researchers often combine these factors to establish isolation and cultivation systems that efficiently obtain target groups while reducing unnecessary costs. Li et al. proposed two isolation and cultivation systems, the Conventional Culture Procedure (CCP) and the Streptomycetes Culture Procedure (SCP), for conducting isolation and cultivation experiments on samples from the Gurbantunggut Desert (Li et al. 2021). CCP and SCP primarily differ in their combination of isolation media and inhibitors. While both strategies yielded significant bacterial isolates, SCP showed superior efficacy in isolating *Streptomyces* strains, highlighting the importance of tailored cultivation systems in achieving specific research objectives.

By focusing on factors such as isolation media composition, inhibitors, and culture conditions, researchers can enhance the precision and efficiency of microbial isolation studies, ultimately leading to a more comprehensive understanding of microbial diversity and ecology.

### Multi-omics-guided isolation and cultivation strategy

**Fig. 2:**
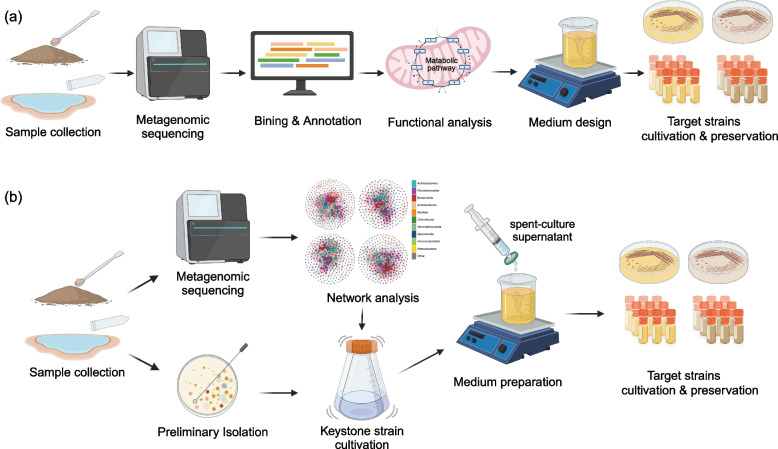
Two multi-omics-guided isolation and cultivation strategies for target strain. (a) cultivation and enrichment of target taxa guided by functional analysis. (b) utilizing network analysis to identify keystone strains for promoting cultivation and enrichment of target taxa

With the advancement of high-throughput sequencing technologies and various algorithms, a deeper understanding of the functional roles of uncultivated microbial communities in various habitats has been achieved through metagenomics and meta-transcriptomics. Targeted isolation and cultivation guided by multi-omics data is considered efficient, especially in extreme environments with low biomass and complex, variable conditions, where specificity is crucial and factors such as microbial interactions and nutritional preferences cannot be overlooked. In a study on microbial diversity in Xizang and Yunnan hot spring sediments, researchers discovered 35 high-quality MAGs representing three novel Actinobacteria clades: *Ca*. Geothermincolia, *Ca*. Humimicrobiia, and *Ca*. Aquicultoria (Jiao et al. 2021). Following 120 days of enrichment, a significant increase in the abundance of the target Actinobacteria suggested the success of the multi-omics-guided enrichment strategy for *Ca.* Geothermincolia (Fig. 2a). Omics-guided enrichment of strains typically yields favorable outcomes when applied to potential functional microbial groups, however, challenges persist in obtaining pure cultures, necessitating the integration of novel technologies such as cell sorting, microfluidics, and membrane diffusion (Lewis et al. 2021).

In another study on microorganisms from Xizang and Yunnan hot spring, Xian et al. identified through OTU co-occurrence network analysis that less abundant OTUs such as the genus *Tepidimonas* (<1% relative abundance) exhibited high centrality (key nodes), while dominant OTUs, particularly the genus *Chloroflexus* (13.9% relative abundance), formed peripheral vertexes (Xian et al. 2020). Leveraging this network analysis, a hypothesis suggesting the growth promotion of *Chloroflexus* strains by *Tepidimonas* strains led to the development of an improved isolation medium containing 10% spent-culture supernatant of *Tepidimonas* sp. strain SYSU G00190W for targeted isolation of *Chloroflexota* spp. strains from the Xizang and Yunnan hot spring samples, resulting in the discovery of several potential novel species (Fig. 2b). Metabolomics studies of the spent-culture supernatant unveiled several low-molecular-weight organic substrates beneficial for the growth of *Chloroflexota* spp. strains, highlighting the crucial role of microbial interactions in strain isolation and cultivation.

### Culturomics strategy

Culturomics is a cultivation method that utilizes various culture conditions, MALDI-TOF mass spectrometry, and 16S rRNA gene sequencing to identify bacterial species (Lagier et al. 2018). Initially employed to isolate and characterize human gut microbiota, microbial culturomics by Lagier et al. identify 1,057 prokaryotic species in human gut samples, including 146 bacterial species known to humans but not previously found in the gut, 187 bacteria not previously isolated from human samples, and 197 potential novel archaeal species, advancing the understanding of the human gut microbiome (Lagier et al. 2016). Microbial isolation and cultivation, particularly in extreme environments, are often facing various challenges, labor- and resource-intensive tasks that may overlook important target taxa within these communities. The concept of culturomics has ushered in a new perspective for microbiological research, especially in culture-dependent studies, leading to continuous refinement and broader applications of culturomics in subsequent developments, such as in the exploration of extremophiles and their genetic resources.

**Fig. 3:**
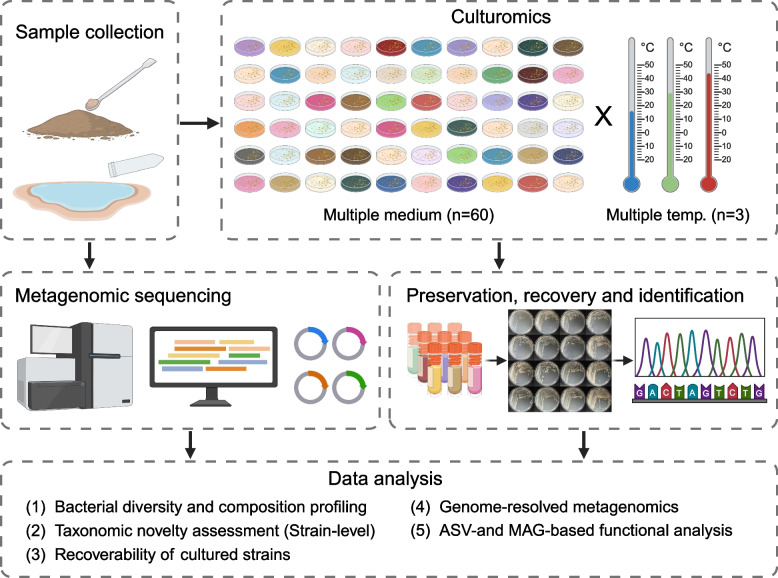
The schematic workflow of Culturomics-Based Metagenomics strategy

However, the high complexity and low biomass of bacterial communities in extreme environments, it is often challenging to capture rare yet crucial microbes in environmental samples through direct metagenomic sequencing (Goodfellow et al. 2018; Li et al. 2021). Furthermore, conventional culturomics methods based on high-throughput MALDI-TOF colony identification may not fully capture all the information from cultures (Huang et al. 2023). In a microbial study of a desert ecosystem, Li et al. introduced culturomics-based metagenomics (CBM) to explore the microbial dark matter of desert ecosystems, integrating large-scale cultivation, full-length 16S rRNA gene amplicon, and shotgun metagenomic sequencing (Fig. 3) (Li et al. 2023).

The research findings indicated that CBM strategy that based on full-length 16S rRNA gene amplicon, recovered more amplicon sequence variants (ASVs) than direct uncultivated sequencing. High-throughput BLAST searches of ASVs against the NCBI 16S rRNA database revealed a substantial number of potential novel taxa (*n*=1095), with the majority being potential novel species (*n*=1007), which far exceeding what was detected through direct sequencing. However, direct sequencing primarily detected potential novel genera, with a total of 398 ASVs, whereas the CBM identified only 88 ASVs belonging to potential novel genera or other higher taxonomic ranks. This illustrates that the CBM strategy can complement direct sequencing to explore environmental microbial resources more comprehensively.

In terms of metagenomics, Li et al. found that the CBM significantly improved the harvestability and assembly quality of MAGs (Li et al. 2023). Applying the CBM achieved 33 high-quality MAGs and 115 medium-quality MAGs from seven cultures. Altogether, 148 MAGs were assigned to two archaeal and 146 bacterial reference classification groups in the Genome Taxonomy Database (GTDB) (Parks et al. 2021). In contrast, direct shotgun metagenomic sequencing with over 30Gb of sequencing depth per desert environmental sample resulted in only one high-quality MAG.

It was noting that Li et al. (2023) succussed to isolate 54 strains covering seven genera from two frozen bacterial stocks obtained through CBM, employing morphological de-duplication methods. Moreover, based on 16S rRNA gene sequence similarity, they identified 4 strains belonging to the phylum *Bacillota* (one strain) and *Actinomycetota* (three strains). This demonstrates that targeted isolation of specific microbes might be achieved via CBM and post hoc recovery approaches (Li et al. 2023).

## Exploration of extremophilic natural product resources

**Fig. 4:**
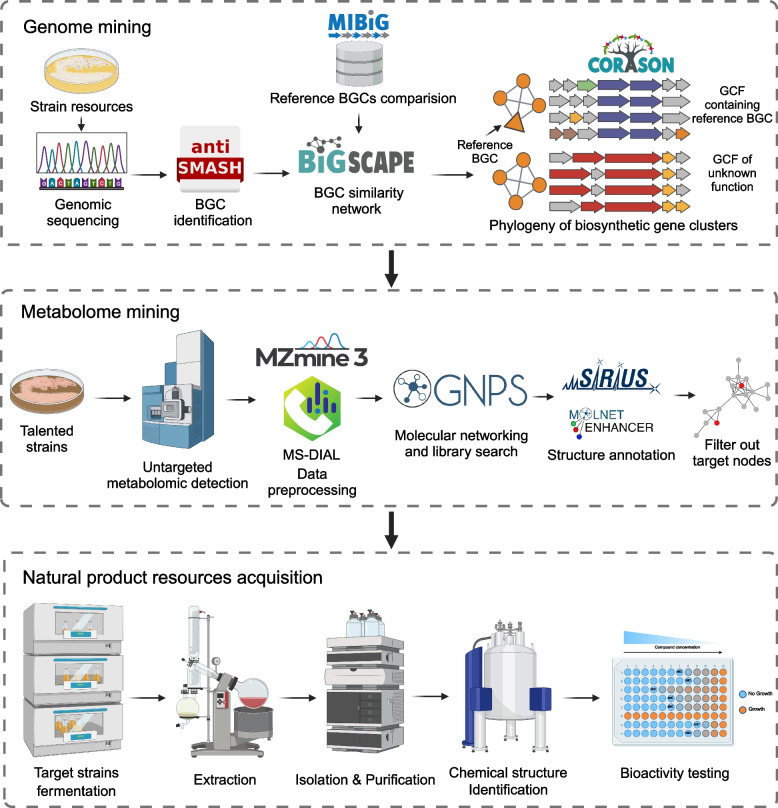
Exploration of natural product resources from extremophilic microorganisms through integrated genomic and metabolomic mining

Environmental conditions play a crucial role in shaping the secondary metabolic capabilities of microorganisms (van Bergeijk et al. 2020). Different habitats exhibit varying climates, geologies, nutrient conditions, and species compositions, leading microorganisms to evolve adaptive survival mechanisms (Medema et al. 2021). Extreme environments like deserts, deep seas, hot springs, and salt lakes exert significant evolutionary pressure on microorganisms residing within them. Microorganisms isolated from these environments often possess unique survival strategies and can produce structurally novel and highly promising natural products (Kohli et al. 2020; Rampelotto 2013), here we call them “metabolic dark matter”. For instance, the Atacama Desert in Chile has been extensively studied, resulting in the isolation of numerous *Streptomyces* spp. strains with novel biosynthetic capabilities. These strains have yielded several natural products with antibacterial and anticancer bioactivities, including Abenquines A-D, Atacamycins A-C, Chaxalactins A-C, Chaxamycins A-D, Chaxapeptin, and Asenjonamides A-C (Abdelkader et al. 2018; Elsayed et al. 2015; Nachtigall et al. 2011; Rateb et al. 2011a; Rateb et al. 2011b; Schulz et al. 2011). Not only aiding in the development of antibiotics in the pharmaceutical field, but microbial natural products also play a crucial role in the industrial sector. Microbial-derived pigments represent a significant direction in the development of the natural pigment industry (Tuli et al. 2015). Characteristics such as widespread distribution, rapid reproduction, diverse species, and ease of genetic modification through bioengineering enable microorganisms to meet market demands for natural pigments. As secondary metabolites, microbial pigments participate in microbial ecological processes. The unique ecological niches provided by extreme environments make microbial resources from these habitats an important source of novel microbial pigments (de Menezes et al. 2023). Additionally, in other high-value industries, ectoine, an amino acid derivative discovered in microorganisms from desert salt lakes, helps microbial cells cope with high osmotic pressure and thermal stress by regulating cellular osmotic balance (Graf et al. 2008). Following its development, ectoine has made a strong entry into the cosmetics industry, with skincare and anti-aging products based on its properties continuously emerging in the market, creating substantial commercial value (Liu et al. 2021). Thus, extremophilic microorganisms represent a rich source of bioactive compounds that can be harnessed to develop products with practical applications for society (Hui et al. 2021). In this section, we introduced the exploration of extremophilic biosynthetic potential using genomic mining and metabolomic mining strategies (Fig. 4). Furthermore, we reviewed the development of exploration strategies for cryptic biosynthetic potential.

### Genome mining

The history of natural product research can be traced back to 1803 when Friedrich Sertürner isolated the morphine entity from *Papaver somniferum* L. (Lockermann 1951). More than two centuries have passed, and we have now entered the genomic era, the application of genome mining technology in natural product-related research not only enhances the discovery rate of natural products but also facilitates the characterization of novel molecular functions and biosynthetic pathways (Medema et al. 2021). The model microorganism *Streptomyces coelicolor* A3(2) has been extensively studied in the field of natural products for the past half-century before its complete genome sequencing, leading to the discovery of approximately a dozen specialized metabolites. With the public availability of the genome sequence, genome mining of *Streptomyces coelicolor* A3(2) has revealed seven different types of metabolites, including the nonribosomal peptides Coelibactin (Bentley et al. 2002) and Coelichelin (Lautru et al. 2005); the sesquiterpene (+)-epi-isozizaene (Lin et al. 2006) and 2-alkyl-4-hydroxymethylfuran-3-carboxylic acids (Corre et al. 2008); the ribosomally synthesized peptide SCO-2138 (Kersten et al. 2011); the polyketide Coelimycin P1(Gomez-Escribano et al. 2012); and a series of novel Arseno-polyketides compounds (Cruz-Morales et al. 2016).

With the popularization of genome sequencing technologies, the genome mining of natural products has rapidly advanced (Table 1). Currently, researchers have developed various powerful computational tools or platforms for uncovering biosynthetic potentials in microbial genomes, such as antiSMASH, PRISM, ARTS, MetaBGC, DeepBGC, RiPPMiner, etc., used for predicting microbial biosynthetic gene clusters (BGCs) (Agrawal et al. 2017; Blin et al. 2023; Hannigan et al. 2019; Mungan et al. 2020; Skinnider et al. 2020; Sugimoto et al. 2019). Among these, antiSMASH is currently the most widely used platform for predicting BGCs. The computational framework Biosynthetic Gene Similarity Clustering and Prospecting Engine (BiG-SCAPE) is capable of constructing protein sequence similarity networks from BGCs predicted by antiSMASH or stored in the MIBiG database, forming gene cluster families (GCFs), determining the priority of BGCs, and facilitating the discovery of new BGCs or GCFs encoding potential novel natural products (Navarro-Muñoz et al. 2020). BiG-SLiCE, functionally similar to BiG-SCAPE, can handle larger datasets (Kautsar et al. 2021). Additionally, experimentally validated BGC databases such as MIBiG, antiSMASH database, IMC-ABC, BiG-FAM, and ClusterMine360 have strongly supported the development and optimization of genome mining computational tools and algorithms (Blin et al. 2020; Kautsar et al. 2020; Mungan et al. 2021; Palaniappan et al. 2019; Terlouw et al. 2022).

**Table 1:** Representative genome mining tools

**BGC prediction**	**Description**	**Reference**
antiSMASH	A web server and stand-alone tool for detecting and characterizing BGCs in archaea, bacteria, and fungi.	(Blin et al., 2023)
PRISM	A comprehensive computational platform for predicting genomically encoded BGCs and antibiotic chemical structures.	(Skinnider et al. 2020)
ARTS	Antibiotic resistant target seeker for comparative genome mining.	(Mungan et al., 2020)
MetaBGC	A computational algorithm for direct detection of small molecule BGCs in complex metagenomic sequencing data of the human microbiome.	(Sugimoto et al., 2019)
DeepBGC	A deep learning genome-mining strategy for BGCs prediction.	(Hannigan et al. 2019)
RiPPMiner	A bioinformatics resource for deciphering the chemical structures of RiPPs through genome mining.	(Agrawal et al. 2017)
BiG-SCAPE	A computational tool for rapid and interactive analysis of sequence similarity networks of BGCs and GCFs.	(Navarro-Muñoz et al. 2020)
BiG-SLiCE	Functionally similar to BiG-SCAPE, capable of processing large volumes of data.	(Kautsar et al., 2021)
**BGC database**		
MIBiG	A repository for microbial BGCs of known function.	(Terlouw et al., 2022)
antiSMASH database	A database for BGCs detected by antiSMASH in publicly available, dereplicated, high-quality microbial genomes.	(Blin et al. 2020)
IMG-ABC	A database of predicted and experimental biosynthetic gene clusters and the secondary metabolites they produce.	(Palaniappan et al., 2019)
BiG-FAM	A database for GCFs.	(Kautsar et al. 2020)
ClusterMine360	A database of microbial polyketide and non-ribosomal peptide gene clusters.	(Conway and Boddy 2012)

With the continuous expansion of whole genome sequences and metagenomic datasets, the rapid development of genome mining tools, and improvements in computational capabilities, researchers are now able to gain deeper insights into microbial biosynthetic potential across global ecological niches. Nayfach et al. collected over 10,000 metagenomes covering global ecosystems, identifying 87,187 potential novel BGCs and indicating that the biosynthetic potential of the phylum *Acidobacteria* may have been underestimated (Nayfach et al. 2021). Paoli et al. studied the biosynthetic potential of global marine microbes through over 1,000 seawater samples, uncovering approximately 40,000 potential novel BGCs from around 10,000 microbial genomes (Paoli et al. 2022). Gavriilidou et al. conducted research on biosynthetic potential using BiG-SLiCE for 170,000 bacterial whole genomes and 47,000 metagenome-assembled genomes (Gavriilidou et al. 2022). This study suggested that only about 3% of bacterially encoded natural products with biosynthetic potential have been experimentally characterized to date, highlighting the phylum *Actinobacteria* as the most diverse biosynthetic lineage among bacteria and revealing that the biosynthetic capabilities of several rare taxa may have been underestimated.

Compared to traditional analytical chemistry techniques, genome mining holds key advantages. Firstly, genome mining can reveal specialized metabolites that microorganisms studied under cultivation conditions do not express, which is considered as cryptic biosynthetic pathways. Moreover, genome studies can provide further theoretical references and understanding for the activation of these cryptic biosynthetic pathways. Secondly, the application of genome mining can link natural product molecules with their corresponding biosynthetic genes, enabling heterologous expression and large-scale production. This is crucial for the development of industries related to biological manufacturing. Thirdly, genome mining requires much less biomass than traditional analytical chemistry research. Since many bioactive molecules are derived from rare or difficult-to-cultivate microbial sources, genome mining significantly reduces sample consumption and research costs.

### Metabolome mining

DNA or RNA sequencing can provide in-depth insights into the biosynthetic potential of microorganisms, while metabolomics directly detects the functional molecules expressed by microorganisms in a system. Therefore, metabolomics is also considered the omics study closest to the phenotype (Bauermeister et al. 2022). Mass spectrometry technology is commonly used as a detection method in metabolomics due to its high sensitivity, low sample requirements, and ability to detect multiple molecules in complex biological samples (Aksenov et al. 2017). Metabolome research started relatively late compared to genomics, and high-throughput and accurate analysis of mass spectrometry data remains challenging. Nevertheless, recent years the development of analyzing tools for metabolomics data based on mass spectrometry are significant increased (Table 2).

**Table 2:** Representative metabolome mining tools

**Data preprocessing**	**Description**	**Reference**
AMDIS	A software designed for the processing of GC-MS data.	(Stein 1999)
MS-DIAL	A universal program for untargeted metabolomics that supports multiple instruments and MS vendors.	(Tsugawa et al. 2015)
MZmine	A scalable mass spectrometry data analysis platform that supports multimodal data analysis for various instrumental setups.	(Schmid et al. 2023)
OpenMS	A software specifically designed for the flexible and reproducible analysis of high-throughput MS data.	(Röst et al. 2016)
XCMS	A software for processing mass spectrometry data for metabolite profiling	(Smith et al. 2006)
MSHub	A software for auto-deconvolution and molecular networking of GC-MS data	(Aksenov et al. 2021)
**Tools for structure annotation**		
MetFrag	*In silico* fragmentation for computer assisted identification of metabolite mass spectra.	(Wolf et al. 2010)
CFM-ID	Competitive fragmentation modeling of ESI- MS/MS spectra for putative metabolite identification.	(Allen et al. 2015)
SIRIUS	Turning MS data into metabolite structure information	(Dührkop et al. 2019)
ZODIAC	Database- independent molecular formula annotation	(Ludwig et al. 2020)
CANOPUS	Predicting compound classes directly from MS/MS	(Dührkop et al. 2021)
Network Annotation Propagation (NAP)	Annotation of analogous compounds in molecular networks	(da Silva et al. 2018)
DEREPLICATOR	Annotation of microbial peptides, or at least a part of the amino acid sequence	(Mohimani et al. 2017)
MS2LDA	Identification of substructures and their co-occurrence in MS/MS datasets.	(van der Hooft et al. 2016)
MolNetEnhancer	Chemical Classification of metabolomic data	(Ernst et al. 2019)

Metabolomics data require a series of preprocessing steps on mass spectrometry raw data before annotation and statistical analysis, including background noise removal, compound identification, ion peak features extraction, and quantification of ion peak features (Tian et al. 2017). Software such as MZmine, MS-DIAL, MSHub, AMDIS, OpenMS, and XCMS can efficiently and accurately preprocess data in high throughput (Aksenov et al. 2021; Röst et al. 2016; Schmid et al. 2023; Tautenhahn et al. 2008; Tsugawa et al. 2015). Following data preprocessing, detected mass spectrometry ion peaks need to be annotated. Mass spectrometry database searches are typically used for annotating known compounds by comparing the MS/MS spectra generated by mass spectrometry detection with reference spectra of known compounds stored in mass spectrometry databases like GNPS, MassBank, NIST, and METLIN (Guijas et al. 2018; Horai et al. 2010; Oberacher et al. 2013; Wang et al. 2016). However, these mass spectrometry databases are limited by the availability of chemical standards, with most MS/MS spectra in databases originating from commercially available chemical standards. Since many microbial molecules are not commercially available, mass spectrometry database searches cannot fully leverage their annotation functionality in microbial metabolomics research. GNPS, as an integrated platform for mass spectrometry data analysis tools, has not only fostered a robust ecosystem of mass spectrometry analysis software but also encourages researchers to upload experimental raw data and processed mass spectrometry datasets. Based on this, GNPS has established the Natural Products Atlas, a molecular mass spectrometry database for microbial metabolites (van Santen et al. 2019). Nevertheless, the current MS/MS reference spectra cover only a small fraction of microbial metabolites, demonstrating limitations in fully supporting metabolomics-related research in the field of metabolite discovery.

To achieve high-throughput annotation of metabolomics data, researchers have developed several algorithmic model-based mass spectrometry annotation tools to overcome the limitations of metabolomics annotation imposed by sole reliance on spectral database searches. MetFrag and CFM-ID predict substructures of MS/MS fragment ions and then combine them based on known chemical bond cleavages for MS/MS spectrum annotation (Allen et al. 2015; Wolf et al. 2010). SIRIUS and ZODIAC software, on the other hand, construct fragment ion trees to generate fingerprint spectra, subsequently matching these fingerprints with small molecule structure databases using machine-learning-trained models (Dührkop et al. 2019; Ludwig et al. 2020). This algorithmic model-based annotation approach generates candidate structure matching lists from molecular structure databases for mass spectrometry data, yielding high-throughput and broad annotation coverage. However, obtaining high-confidence unique matching structures remains challenging, necessitating cautious interpretation of annotation results. CANOPUS is a computational tool for systematic compound class annotation in nontargeted tandem mass spectrometry metabolomics, utilizing a deep neural network to predict compound classes from fragmentation spectra, thereby enabling analysis of molecules lacking spectral or structural reference data and advancing the understanding of biological systems and chemical diversity at the compound class level (Dührkop et al. 2021). The Network Annotation Propagation (NAP) enhances computational predictions of unknown fragmentation mass spectra in untargeted mass spectrometry analysis by leveraging molecular networking to propagate structural annotations (da Silva et al. 2018). NAP improves annotation accuracy through network consensus and re-ranking of candidate structures based on molecular network topology and similarity, even in instances where there is no match to MS/MS spectra in standard libraries. MS2LDA is an unsupervised method inspired by text-mining techniques that is designed to extract common patterns of mass fragments and neutral losses, termed Mass2Motifs, from sets of fragmentation spectra (van der Hooft et al. 2016). By structurally characterizing these Mass2Motifs, the algorithm enables the annotation of molecules lacking reference spectra and reveals biochemical relationships between different molecules. DEREPLICATOR is a deduplication algorithm enabling high-throughput peptide natural product identification and interoperability with large-scale mass spectrometry-based screening platforms for natural product discovery (Mohimani et al. 2017). MolNetEnhancer is a workflow integrating outputs from molecular networking, MS2LDA, computational annotation tools (such as NAP or DEREPLICATOR), and automated chemical classification through ClassyFire, to offer a more comprehensive chemical overview of metabolomics data while elucidating the structural details of each fragment spectrum (Ernst et al. 2019).

### Exploration of cryptic biosynthetic potential

In industrial and laboratory settings, microorganisms are typically cultivated in nutrient-rich and controlled artificial environments. This practice significantly diverges from the complex interactions and varied climatic conditions of their natural habitats. Consequently, the comprehensive expression of the biosynthetic potential of microorganisms remains elusive, representing a substantial challenge in contemporary natural product research (Atanasov et al. 2021).

Recognizing the vital role of microbial secondary metabolites in their survival and ecological niche establishment (Wright and Vetsigian 2016), microbiologists have asserted that interspecies microbial interactions are crucial for the activation of silent BGCs (Abrudan et al. 2015). To activate silent BGCs under experimental conditions, several studies have simulated the "chemical-ecological" relationships found in natural ecosystems. For example, Onaka et al. activated silent BGCs and obtained the antibiotic Alchivemycin A by co-cultivating *Streptomyces* sp. S-522 with *Tsukamurella pulmonis*, a bacterium capable of producing lanthipeptides (Onaka et al. 2015). Similarly, Sung et al. reported an increase in antibiotic production and enhanced biological activity in the fermentation broth by co-cultivating marine actinomycetes with drug-resistant *Staphylococcus aureus*, among other human pathogens (Sung et al. 2017). The triggering factors for microbial metabolic network regulation are diverse, encompassing physical interactions between cells, variations in nutrient depletion rates, enzyme-catalyzed reactions of compound precursors, horizontal gene transfer, and microbial small molecule interactions (Ezaki et al. 1992; Kurosawa et al. 2008; Onaka et al. 2015; Pérez et al. 2011; Traxler et al. 2013). Traditionally, methods for manipulating microbial metabolic lineages based on this theory include altering the nutrient composition of the culture medium, antibiotic resistance induction, and co-culturing strategies (Bode et al. 2002; Hosaka et al. 2009; Hoshino et al. 2015). With advancements in research technologies, several new natural product exploration strategies based on this theory have emerged. Xu et al. proposed a universal high-throughput method for activating silent BGCs in various microorganisms (Xu et al. 2019). By utilizing elicitor screening and imaging mass spectrometry under approximately 500 conditions, this approach revealed novel secondary metabolites from bacteria, including the discovery of nine cryptic metabolites with potential therapeutic bioactivities, such as a new glycopeptide chemotype with potent antiviral properties. Another study demonstrated the identification of genes co-evolved with BGCs in *Streptomyces* strains through phylogenomic analysis (Wang et al. 2024). Engineering the pyrroloquinoline quinone gene cluster into multiple strains resulted in significant enhancements in metabolite production, including known natural products and activated silent BGCs, thereby showcasing a novel and universal strategy to boost polyketide productivity.

With the continual advancement of biochemical technologies, researchers have begun employing genome editing techniques to activate silent BGCs, aiming to overcome challenges in natural product research. Over the past several years, scientists have developed various versatile heterologous hosts for expressing novel drug leads discovered in disparate microorganisms. Enghiad et al. introduced CAPTURE, a scalable direct cloning method that utilizes Cas12a and Cre-lox recombination, enabling efficient cloning of large and complex BGCs (Enghiad et al. 2021). This approach facilitates high-yield discovery of novel bioactive compounds. It was exemplified by the successful isolation of 15 previously uncharacterized natural products, including potent antimicrobials against various pathogens. CAT-FISHING (CRISPR/Cas12a-mediated fast direct biosynthetic gene cluster cloning) presented a rapid and effective method for capturing large BGCs directly from microbial genomes (Liang et al. 2022). CAT-FISHING efficiently captured diverse BGCs, including a 145 kb GC-rich cluster. This enabled the discovery and heterologous expression of a novel anticancer compound, marinolactam A, showcasing its utility in natural product-based drug discovery. Libis et al. established CONKATseq, a targeted sequencing method for identifying physically clustered biosynthetic domains in complex soil metagenomes, revealing many uncharacterized gene clusters from rare soil microbial populations, typically overlooked by conventional sequencing methods (Libis et al. 2019). Subsequently, in a study involving 100 *Streptomyces* strains, CONKATseq was employed to efficiently localize clones carrying intact BGCs as candidates for heterologous expression. This led to the discovery of an antibiotic active against multidrug-resistant *Staphylococcus aureus* (Libis et al. 2022).

In recent years, the field of Artificial Intelligence (AI) has witnessed rapid development, emerging as a highly prominent research focus. The advancement of AI has also permeated the realm of natural product research, ushering in an era where the discovery of natural product-based drugs is transitioning towards computer-aided AI identification and evaluation (Saldívar-González et al. 2022). Previous research teams have utilized deep learning and neural networks to forecast antibiotics with structures distinct from known small molecules, leading to the discovery of the compound Halicin, which exhibited potent antimicrobial effects against drug-resistant bacteria in a mouse model (Stokes et al. 2020). Das et al. integrated deep generative classifiers with molecular dynamics models to design and synthesize 20 potential antimicrobial peptides within 48 days (Das et al. 2021). While AI-driven molecular design models enable rapid exploration of vast chemical spaces, challenges in synthesizing the generated molecules persist. Consequently, Swanson et al. developed the SyntheMol generation model, which enabled the design of easily synthesizable novel compounds from a chemical space of nearly 30 billion molecules. They successfully applied SyntheMol to design molecules inhibiting *Acinetobacter baumannii*, ultimately experimentally validating six structurally novel and biologically active compound molecules (Swanson et al. 2024).

It is evident that the exploration of cryptic biosynthetic potential is a significant and broad scientific challenge, necessitating interdisciplinary collaboration to be effectively addressed. The discussion above has outlined three directions for natural product exploration, highlighting the importance of interdisciplinary backgrounds in advancing research in this field. While researchers from multiple disciplines have been engaged in natural product discovery, leveraging their respective expertise to propel progress in this scientific inquiry, numerous challenges and bottlenecks persist in this area. However, with the continuous expansion of extremophile single-cell and metagenomic databases, ongoing advancements in microbiological theoretical research, further development of gene editing tools, iterative enhancements in biochemical analysis technologies, and the continual progress in computer software and hardware, natural product development and application based on biosynthetic potential will continue to make significant contributions to human health and societal development. Furthermore, the exploration and application of extremophilic microbial resources are poised to advance further synthetic biology study and bio-manufacturing industry.

## Conclusion and future remarks

Decades of diversity research have illuminated the extensive phylogenetic breadth of microbial communities in Earth's extreme environments. Additionally, the accelerated reconstruction and characterization of genomes from novel microorganisms in these environments have significantly reshaped the tree of life and profoundly altered our understanding of archaea and bacteria (Shu and Huang 2022).

However, metagenomic sequencing is not a flawless methodology for microbial ecology studies. These culture-independent techniques facilitate the efficient exploration of community species composition and ecological functions, but they inevitably result in certain omissions (New and Brito 2020). Despite the prevailing belief that traditional culture techniques fail to cultivate 99% of microbes, recent studies combining culture-dependent and culture-independent methods, especially in extreme environments, suggest that this ratio might be overestimated (Li et al. 2021; Li et al. 2023). Culture-dependent diversity investigation methods may distort the representation of microecology due to enrichment effects. We propose that integrating culture-dependent and culture-independent approaches is rational for deepening our understanding of microecology in extreme environments, validating physiological and ecological functions, and exploring microbial resources. Notable examples of successful integration include multi-omics-guided isolation and cultivation strategies, and CBM strategies (Li et al. 2023; Yang et al. 2023).

Furthermore, the exploration of natural product resources from extremophiles also benefits from integrated omics strategies. Genomic mining has revealed the vast biosynthetic potential of microbes, yet many biosynthetic pathways remain cryptic. Metabolomic mining provides clear insights into the metabolites produced by these organisms. However, the high-throughput analysis of untargeted mass spectrometry-based metabolomics data remains a significant challenge (Bauermeister et al. 2022). The integration of genomics and metabolomics represents a substantial technological advancement for discovering microbial natural products and investigating microbial niches and interactions (Bauermeister et al. 2022; van der Hooft et al. 2020). This integration is also a key area for computational biology.

Therefore, the importance of microbial resources in extreme environments is undeniable. Investigating, developing, and utilizing these resources necessitates the integration of multiple omics and strategies. Addressing the technical challenges in this integration requires not just efforts from individual laboratories, but a collaborative effort from the entire research community. Sharing knowledge and developing systematic, reproducible analytical pipelines and cooperative networks are essential for progress in this field.
